# Characterisation of the main PSA glycoforms in aggressive prostate cancer

**DOI:** 10.1038/s41598-020-75526-3

**Published:** 2020-11-04

**Authors:** Anna Gratacós-Mulleras, Adrià Duran, Akram Asadi Shehni, Montserrat Ferrer-Batallé, Manel Ramírez, Josep Comet, Rafael de Llorens, Radka Saldova, Esther Llop, Rosa Peracaula

**Affiliations:** 1 Biochemistry and Molecular Biology Unit, Department of Biology, University of Girona, C/Maria Aurèlia Capmany 40, 17003 Girona, Spain; 2 Girona Biomedical Research Institute (IDIBGI), Girona, Spain; 3 GlycoScience Group, National Institute for Bioprocessing Research and Training (NIBRT), Fosters Avenue, Mount Merrion, Blackrock, Co Dublin Ireland; 4 Clinic Laboratory, Dr. J. Trueta University Hospital, Girona, Spain; 5 Urology Unit, Dr. J. Trueta University Hospital, Girona, Spain; 6 UCD School of Medicine, College of Health and Agricultural Science (CHAS), University College Dublin (UCD), Dublin, Ireland

**Keywords:** Analytical biochemistry, Carbohydrates, Prostate cancer

## Abstract

Serum levels of prostate specific antigen (PSA) are commonly used for prostate cancer (PCa) detection. However, their lack of specificity to distinguish benign prostate pathologies from PCa, or indolent from aggressive PCa have prompted the study of new non-invasive PCa biomarkers. Aberrant glycosylation is involved in neoplastic progression and specific changes in PSA glycosylation pattern, as the reduction in the percentage of α2,6-sialic acid (SA) are associated with PCa aggressiveness. In this study, we have characterised the main sialylated PSA glycoforms from blood serum of aggressive PCa patients and have compared with those of standard PSA from healthy individuals’ seminal plasma. PSA was immunoprecipitated and α2,6-SA were separated from α2,3-SA glycoforms using SNA affinity chromatography. PSA *N-*glycans were released, labelled and analysed by hydrophilic interaction liquid chromatography combined with exoglycosidase digestions. The results showed that blood serum PSA sialylated glycoforms containing GalNAc residues were largely increased in aggressive PCa patients, whereas the disialylated core fucosylated biantennary structures with α2,6-SA, which are the major PSA glycoforms in standard PSA from healthy individuals, were markedly reduced in aggressive PCa. The identification of these main PSA glycoforms altered in aggressive PCa opens the way to design specific strategies to target them, which will be useful to improve PCa risk stratification.

## Introduction

Prostate cancer (PCa) is the most common carcinoma among men worldwide and it is the second most frequent cause of cancer death in men in western countries^1^.

PCa diagnosis is based on a combination of digital rectal examination (DRE) and measurements of prostate specific antigen (PSA) blood serum levels^2^. Even though the monitoring of PSA levels is a non-invasive tool in the diagnosis of patients with prostatic pathologies, it is not specific enough for PCa. Indeed, serum PSA levels could be also elevated in benign prostate disorders such as benign prostate hyperplasia (BPH) or prostatitis^3^. For this reason, biopsies must be performed to distinguish benign prostate alterations from PCa. However, only 25% of biopsies with elevated serum PSA are PCa, and many of them are indolent cancers^4,5^. This leads to increase in false-positive results, misdiagnosis and overtreatment of patients^6,7^. Among men with localised PCa of low or intermediate risk, surgery has not significantly demonstrated higher PCa survivals than PSA follow up, but it has been associated with higher frequency of adverse side effects, such as urinary incontinence and erectile dysfunction. Currently, PCa aggressiveness is classified by histopathological analysis, which means the need of performing biopsies. Therefore, non-invasive biomarkers with high sensitivity and specificity to diagnose patients with PCa are required. These should differentiate aggressive cancers from the slow growing ones in order to help in the clinical decision process and assist the personalised treatments^6,8,9^.

PSA is a serine protease and belongs to the human kallikrein family. Most of the PSA in blood circulation is bound to protease inhibitors and only a low percentage is present as free PSA (fPSA). The proteolytic activity of PSA in blood is inhibited by the formation of a PSA complex mainly with α1-antichymotrypsin (ACT) or in a minor quantity to α2-macroglobulin (A2M) or α1-antitrypsin^10^. The rest of PSA forms (free forms) are proteolytically inactive. Blood serum PSA screening is based on fPSA and total PSA (tPSA) forms quantification. This fPSA includes the precursor PSA isoforms, internally cleaved PSA benign forms and inactivated but not cleaved PSA forms^11^, and tPSA comprises fPSA forms and the PSA complexed with the inhibitors. PSA-ACT complex in blood serum is much higher in patients with PCa than in BPHs^12–15^. The ratio of fPSA to tPSA has been used to improve the PCa detection and the values lower than 0.25 are indicative of PCa. However, the free/total PSA ratio as a PCa biomarker has been questioned for its benefit due to its high false-positive rate in the diagnosis^7,16^. In order to improve PCa diagnosis, PSA kinetics, PSA density and velocity have also been used, nonetheless they are not good predictors of aggressiveness^17^. Other biomarkers such as the prostate health index (PHI), the prostate cancer antigen 3 (PC3) and the 4 K score have shown to improve the specificity to differentiate benign prostate alterations and PCa, however, they are not specific enough to distinguish indolent from aggressive PCa^18–21^.

Glycosylation is a common post-translational modification of proteins. Changes in the glycosylation pattern of glycoproteins have been found in cancer conditions, which are related to the tumour development and progression^22,23^. Alterations in the glycosylation pattern have been reported in PCa^24,25^. In particular, PSA, which is a glycoprotein with one *N*-glycosylation site at asparagine 69, has shown changes in its glycosylation pattern in PCa cell lines and blood serum from PCa patients compared to PSA from seminal plasma of healthy controls (from now on to be referred to as a standard PSA, in this paper). Specifically, PSA presents changes in core fucosylation, sialylation and *N*-acetylgalactosamine (GalNAc in glycoforms containing GalNAcβ1-4GlcNAc (LacdiNAc) structure) proportions, which are closely associated with aggressive PCa^26–34^. To determine the PSA glycosylation pattern, specific lectins, which are carbohydrate binding proteins, have been used^35^. *Pholiota squarrosa* (PhoSL), *Sambucus nigra* agglutinin (SNA) and *Wisteria floribunda* agglutinin (WFA) lectins have high affinity for core fucose, α2,6-sialic acid (SA) and GalNAc residues, respectively^36–38^. As the most abundant PSA form in blood is complexed with ACT, some researchers have released PSA from ACT by ethanolamine treatment to increase PSA levels and to perform a comprehensive glycosylation analysis^27,28,39^. The PSA *N*-glycans from both fPSA and PSA-ACT complex of PCa blood serum samples have been reported to present the same glycan profile^40^.

Standard PSA has a high percentage of α2,6-SA, about 75%, and around 70–80% of PSA *N*-glycans are core fucosylated^29,41^. Moreover, the major glycoforms are sialylated biantennary structures, being the α2,6-sialylated core fucosylated the predominant ones^27^.

A significant decrease in the percentage of α2,6-SA PSA glycoforms in blood serum PSA from high-risk PCa patients compared to BPH or indolent PCa using SNA affinity chromatography has been reported^27,28,32^. SNA affinity chromatography separated the α2,3-SA from α2,6-SA of PSA glycoforms in two different fractions. However, each of these fractions contained several PSA glycoforms. Those particular PSA glycoforms that are specifically increased or decreased in each of these fractions in the aggressive PCa patients are still unknown, and could have potential to discriminate the aggressive PCa.

The aim of this study is to perform a structural analysis of the PSA glycans from the fractions containing α2,3- and α2,6-SA glycoforms after SNA chromatography from aggressive PCa blood serum samples and to identify the particular PSA glycoforms that are mostly altered compared to standard PSA. Our results have shown noteworthy alterations in the sialic acid linkage and GalNAc content in the main PSA glycoforms of aggressive PCa. In particular, we found an important decrease of the disialylated core fucosylated biantennary structures with α2,6-SA (FA2G2S2 α2,6/α2,6 and FA2G2S2 α2,6/α2,3) and an increase of the α2,3-disialylated core fucosylated biantennary (FA2G2S2 α2,3/α2,3) and α2,3-monosialylated non-core fucosylated (A2G2S1 α2,3) structures. In addition, results showed a rise of GalNAc glycoforms, in α2,3/α2,6-disialylated core fucosylated biantennary chains (FA2G1GalNAc1S2 α2,3/α2,6 isomers) and in α2,6-monosialylated structure (FA2G1GalNAc1S1 α2,6). Some of the aggressive PCa samples also displayed an increase in the α2,3-monosialylated non-core fucosylated biantennary structure (A2G1GalNAc1S1 α2,3).

## Results

### Selected cohort of aggressive PCa patients

To perform a comprehensive analysis of the altered PSA glycoforms associated with aggressive PCa, we selected six patients with aggressive PCa with high levels of blood serum PSA (over 300 ng/ml) to obtain micrograms of pure PSA for each patient to perform its *N*-glycan characterisation. To characterise the glycan structures containing the α2,3-SA and α2,6-SA from the PSA glycoforms, SNA affinity chromatography was performed to separate the PSA glycoforms with α2,6-SA from those with α2,3-SA or neutral structures.

### Characterisation of standard PSA glycoforms by hydrophilic interaction liquid chromatography on ultra-performance liquid chromatography (HILIC-UPLC)

The first aim was to fully characterise the glycoforms of standard PSA to facilitate the latter identification of these structures in the PSA *N*-glycan profiles from the fractions obtained after the SNA affinity chromatography in PCa blood serum samples (explained in the next section).

**Figure 1:**
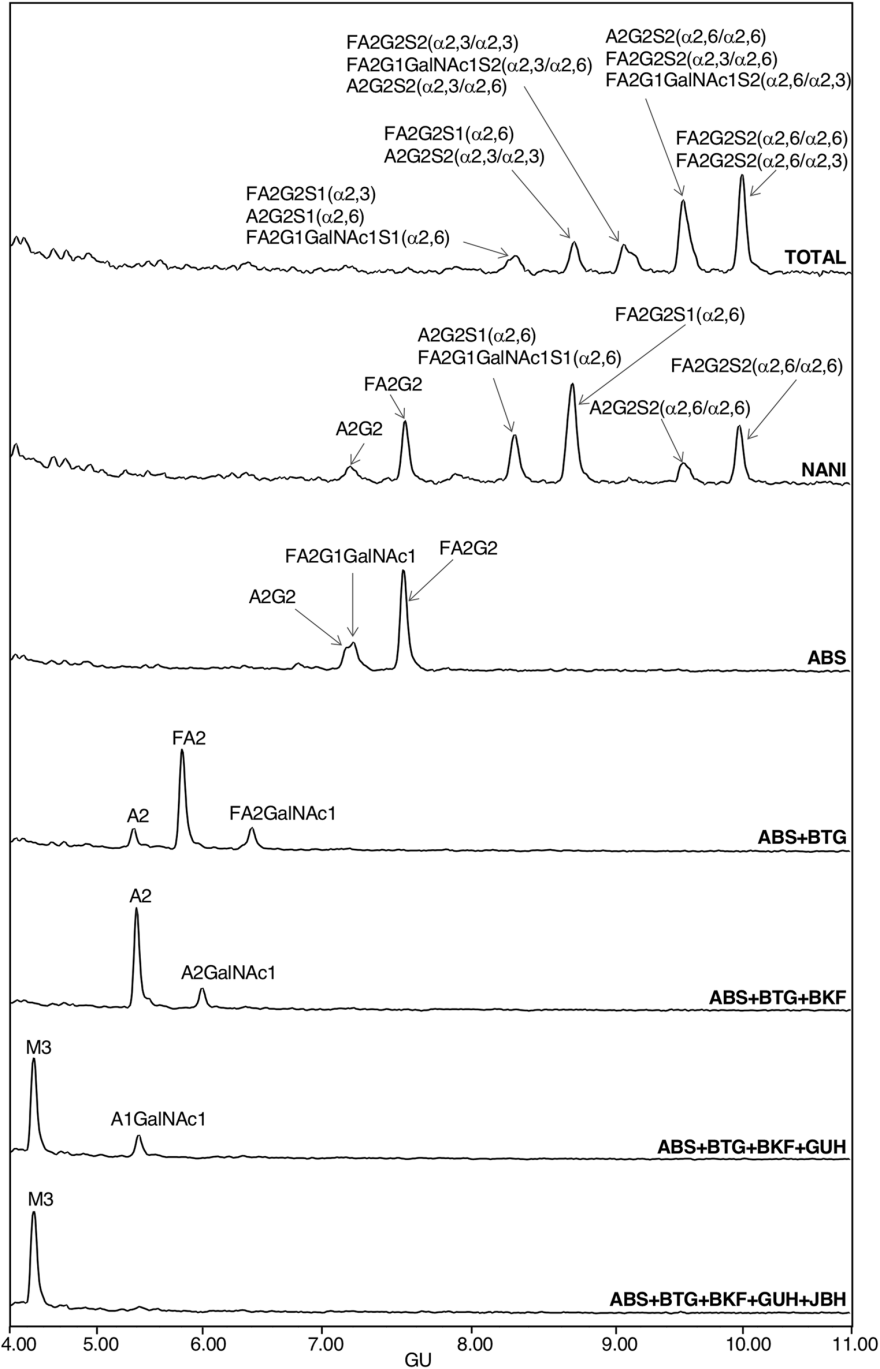
HILIC-UPLC profiles of standard PSA *N*-glycans labelled with 2AB. From top to bottom, chromatograms of consecutive panels correspond to the total profile and the corresponding digestions by the specified exoglycosidases. Profiles are standardised against a dextran hydrolysate with glucose units (GU). Abbreviations used for the different structures are defined in Table 1.

Standard PSA was resolved by SDS-PAGE and PSA *N*-glycans were released and analysed by HILIC-UPLC combined with an array of specific exoglycosidases (NAN1, ABS, BTG, BKF, GUH and JBH). The *N*-glycans were assigned based on their retention time converted to glucose units (GUs) according to the GlycoStore database (https://glycostore.org/search) and by comparing their profiles after performing the exoglycosidase digestions^42^. These results are represented in Fig. 1 and Tables 1 and 2. Relative quantification of each glycan peak was calculated by dividing the area of the corresponding glycan peak by the total area of *N*-glycan peaks in the profile.

**Table 1:**
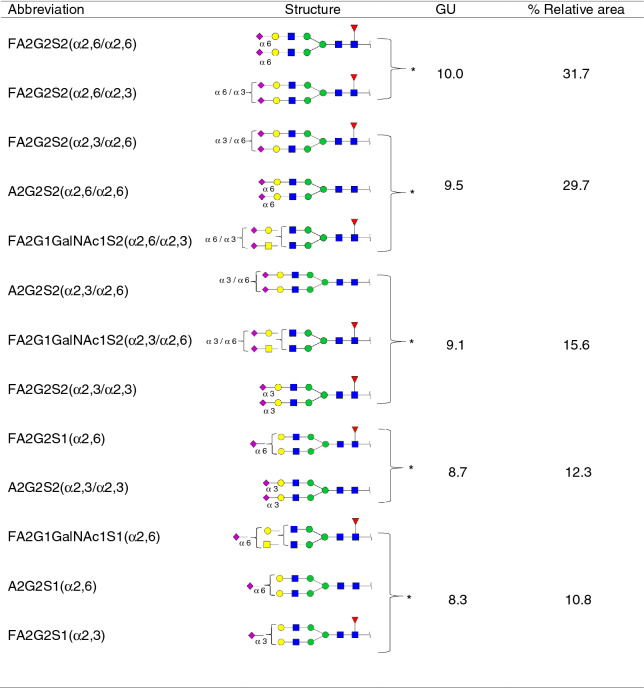
Characterised glycans from standard PSA from total HILIC-UPLC profiles.

Co-eluted glycans are shown with a square bracket with an asterisk.

**Table 2:**
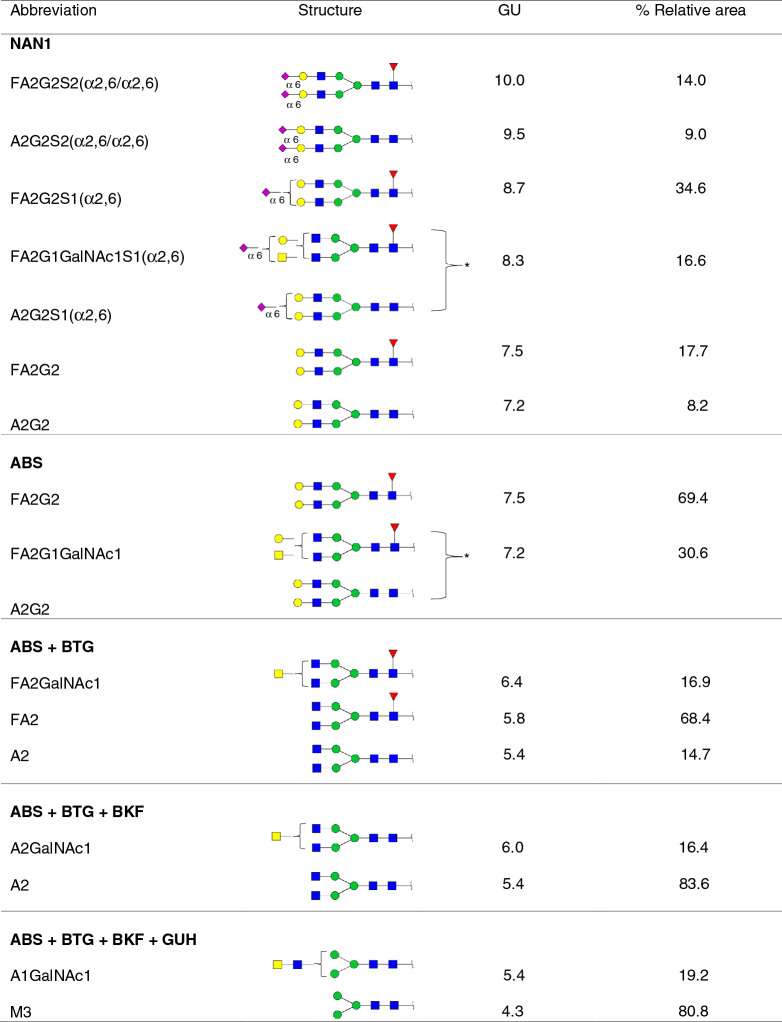
Characterised glycans from standard PSA from digested HILIC-UPLC profiles.

Co-eluted glycans are shown with a square bracket with an asterisk.

In agreement with previously published data^27,29,32^ we confirmed by NAN1 treatment (which releases α2,3-SA) that around 26% of standard PSA glycoforms only contained α2,3-sialylated glycans (calculated from the percentage of FA2G2 and A2G2 obtained after this digestion) (Table 2). Approximately 70% of them were core fucosylated. Moreover, ABS treatment digested all sialylated structures to neutral ones, corroborating that all the PSA glycan structures identified contained SA (Fig. 1).

The percentages of the PSA structures containing core fucose and GalNAc were calculated from the ABS + BTG and ABS + BTG + BKF digestions. BTG digests outer galactoses but not *N*-acetylgalactosamines, and BKF digests fucoses. As a result, around 85% of the *N*-glycans were core fucosylated and nearly 17% contained LacdiNAc, instead of LacNAc (Galβ1-4GlcNAc). The presence of LacdiNAc structures was further confirmed by comparing ABS + BTG + BKF + GUH and ABS + BTG + BKF + GUH + JBH digestions. GUH digests outer *N*-acetylglucosamines but not *N*-acetylgalactosamines, while JBH digests all outer *N*-acetylhexosamines. Only the product from the latter yielded the pentasaccharide core of the *N*-glycans (Manα1–6(Manα1–3)Manβ1–4GlcNAcβ1–4GlcNAc), thus corroborating the presence of the GalNAc residues. All the characterised structures are summarised in Table 2.

### SNA chromatography from aggressive PCa blood serum samples and standard PSA

Standard PSA was used as a control and was subjected to the same experimental steps than the ones for the aggressive PCa blood serum samples. Standard PSA was spiked into pooled female blood sera (without endogenous PSA) as comparable matrix to the blood serum PCa samples.

The PSA purification steps before *N*-glycan sequencing were: (a) releasing the PSA bound to ACT from blood serum samples; (b) immunoprecipitation of the blood serum tPSA; (c) desalting and PSA concentration; (d) SNA affinity chromatography to separate the unbound (UB) and bound (B) fractions of PSA containing α2,3- and α2,6-SA glycoforms, respectively, and tPSA quantification of each fraction by ELECSYS immunoassay; (e) further immunoprecipitation of the PSA from the UB and B fractions with anti-fPSA antibodies; and (f) gel electrophoresis followed by Coomassie staining of the immunoprecipitated samples to detect the purified blood serum PSA bands in the SNA UB and B fractions.

Concerning the PSA proportion of the UB and B fractions of each analysed sample (step d), the UB fraction of the standard PSA (corresponding to α2,3-SA glycoforms) represented 23% of the total PSA (Table 3), in agreement with the 26% that resulted from the *N*-glycan sequencing analysis previously performed with the standard PSA (see Table 2). On the other hand, the comparison of the UB percentages of standard PSA and PSA from aggressive PCa samples showed that the former had a lower proportion of α2,3-SA (23%), while the latter was higher than 30% (32.4–90.2%) in all the blood serum aggressive PCa samples (Table 3). These results are in agreement with previously reported data^27,28^, where a threshold ≥ 30% of PSA in the UB fraction was indicative of aggressive PCa with high sensitivity and specificity.

**Table 3:** Relative percentage of fPSA containing α2,3 sialic acid glycoforms from the unbound (UB) fractions of prostate cancer samples after SNA-lectin chromatography.

Sample ID	% of fPSA containing α2,3-SA (UB fraction)
Standard PSA	22.6
PCa1	32.4
PCa2	44.9
PCa3	61.0
PCa4	90.2
PCa5	35.1
PCa6	41.1

**Figure 2:**
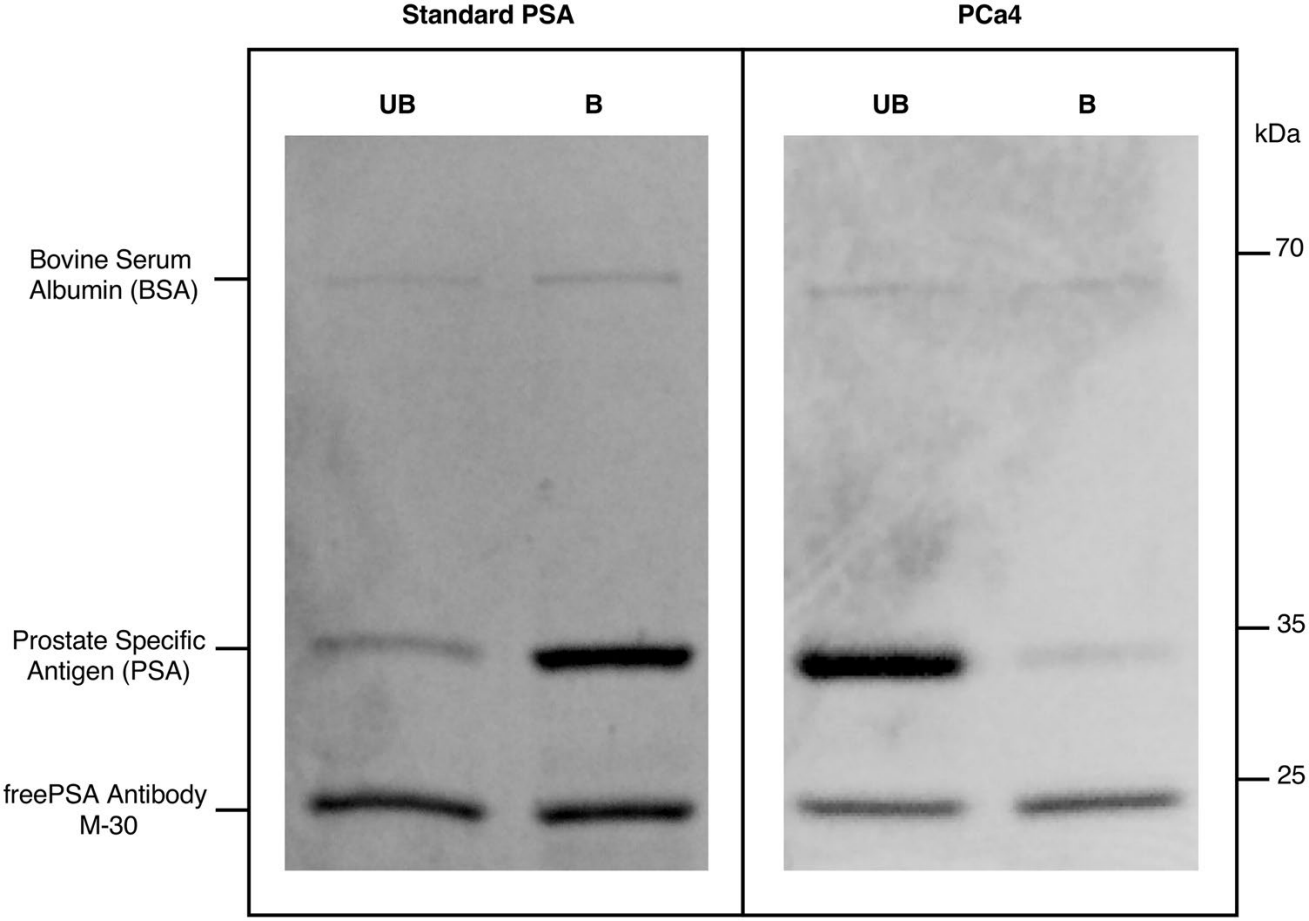
Representative gel electrophoresis of fPSA immunoprecipitated from unbound (UB) and bound (B) fractions of SNA affinity chromatography. Results of standard PSA and PSA from prostate cancer (PCa4) corresponding to two different gels are shown.

To confirm that the protein bands at the molecular weight of 36 kDa (step f) only contained PSA (Fig. 2, suppl. Fig. 1 and suppl. Fig. 2), Matrix-Assisted Laser Desorption/Ionization Time-of-Flight Mass Spectrometry (MALDI-TOF–MS) analyses of the trypsin digested protein bands after PNGase F digestion, which releases *N*-glycans, were performed with the sample of standard PSA spiked in female sera. Peptide mass fingerprinting confirmed that PSA was the only protein present in the gel bands. Eight peptides of PSA (position 25–33, 34–45, 48–68, 71–77, 78–107, 110–125, 195–201 and 246–250) were detected, which represented protein sequence coverage of 45%, with a protein score of 124.

The relative quantification of the UB and B PSA bands was performed by densitometry. An increase of the PSA UB fraction was found in the aggressive PCa samples compared to the corresponding UB fraction of standard PSA (Fig. 2). The quantification of the PSA bands by densitometry showed practically the same relative percentages of PSA in the UB and B SNA fractions that had previously been quantified by ELECSYS (shown in Table 3).

### *N*-glycan sequencing of PSA glycans from the unbound and bound SNA fractions of aggressive PCa and standard PSA

**Figure 3:**
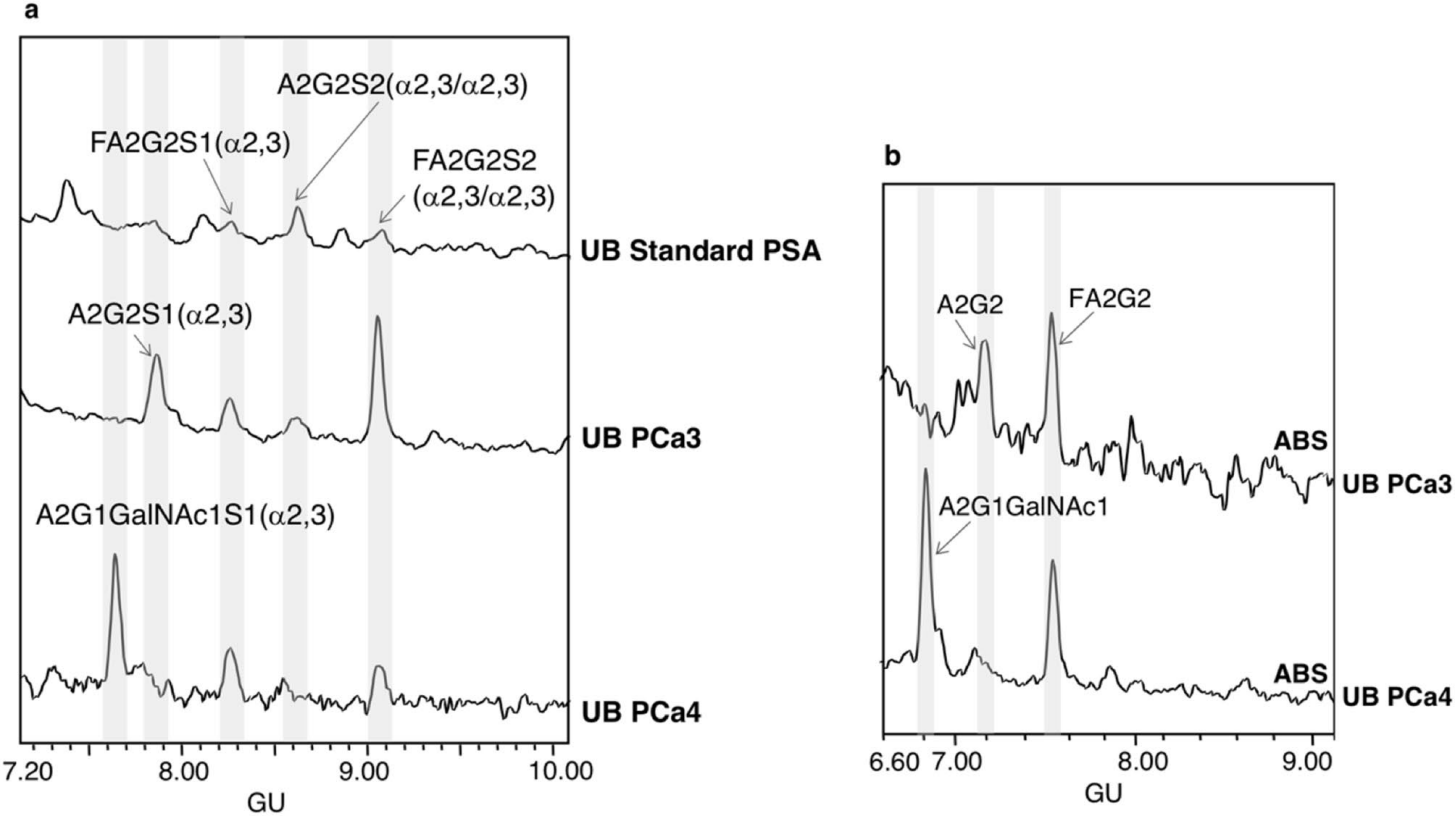
HILIC-UPLC profiles of PSA *N*-glycans from unbound (UB) fractions. (**a**) Total UB fractions of standard PSA (top panel) and PSA from aggressive prostate cancer (PCa3-PCa4) (middle and bottom panel) and (**b**) ABS digested *N*-glycans from PSA from UB fractions of PCa3 (top panel) and PCa4 (bottom panel). Profiles are standardised against a dextran hydrolysate with glucose units (GU).

**Figure 4:**
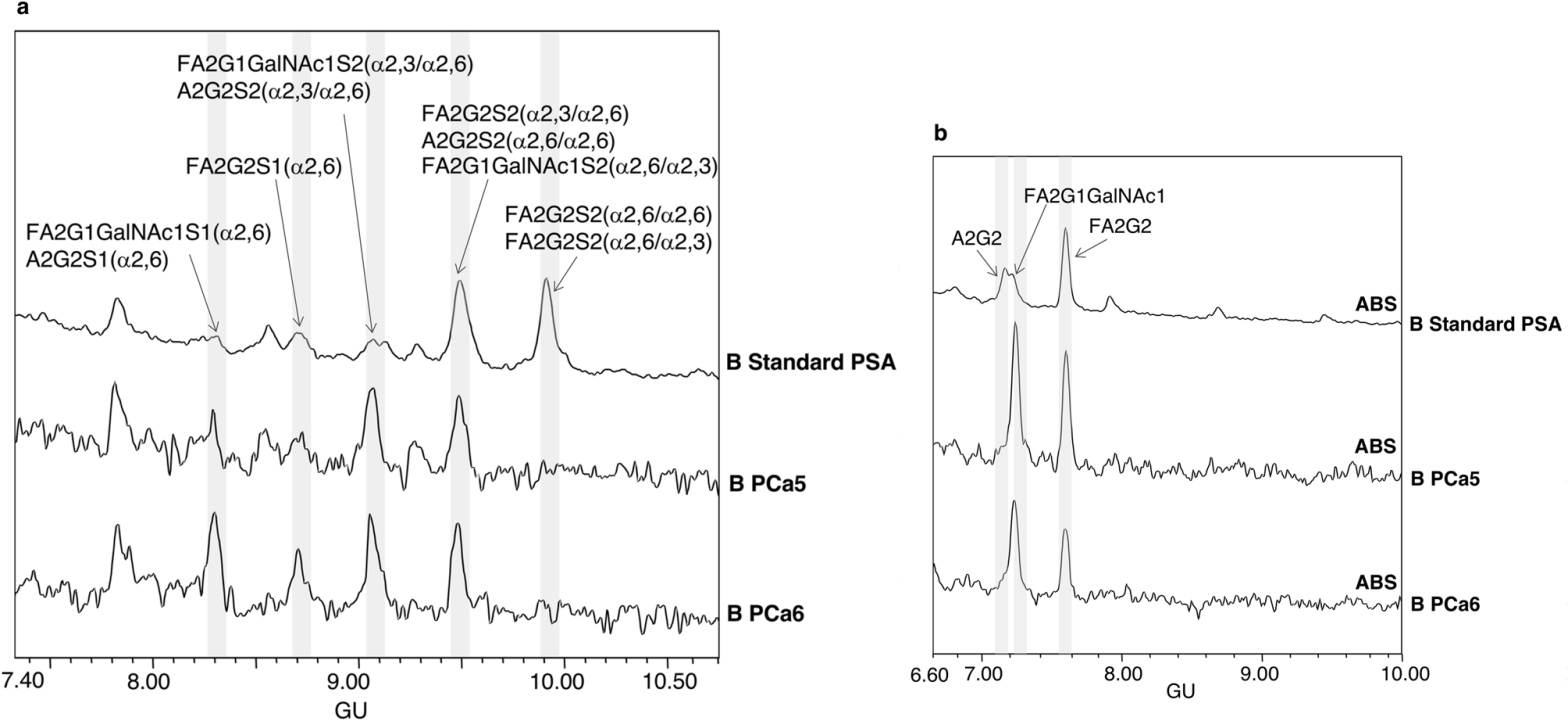
HILIC-UPLC profiles of PSA *N*-glycans from bound (B) fractions. (**a**) Total B fractions of standard PSA (top panel) and PSA from aggressive prostate cancer (PCa5-PCa6) (middle and bottom panel) and (**b**) ABS digested *N*-glycans of B fraction from standard PSA (top panel), PCa5 and PCa6 (middle and bottom panel). Profiles are standardised against a dextran hydrolysate with glucose units (GU).

*N*-glycans were released from the pure PSA bands of UB and B fractions of aggressive PCa and standard PSA samples and characterised by *N*-glycan sequencing. Their structures were compared among all PCa1-6 blood serum samples’ patients and with those obtained from the standard PSA (Figs. 3 and 4).

Regarding the UB profiles (Fig. 3), four PSA *N*-glycans with α2,3-SA were assigned in the standard PSA in agreement with the total *N*-glycan pattern previously determined (Fig. 1). The signal of this profile was quite low and precluded further quantification of the peaks. The UB profiles of the six aggressive PCa blood serum samples showed two different pattern groups (Fig. 3a). From both patterns, PCa3 and PCa4 profiles are representative of four and two PCa samples, respectively. Group 1 pattern, characteristic of PCa1, PCa2, PCa3 and PCa5 samples, showed an increase of FA2G2S2 (α2,3/α2,3) and A2G2S1 (α2,3) glycans compared with the standard PSA *N*-glycans, whereas group 2 pattern (including profiles from PCa4 and PCa6 samples) showed a decrease in the A2G2S1 (α2,3) glycoform and presented the predominant glycoform A2G1GalNAc1S1 (α2.3) at 7.7 GU. This last glycoform with a GalNAc residue was not detected in standard PSA or in group 1 PCa blood serum samples. The percentage of structures containing GalNAc residues were calculated from the profiles obtained after ABS digestion, which releases SA. The two *N*-glycan peaks obtained after ABS digestion in group 2 PCa blood serum samples corresponded to a non-fucosylated biantennary chain with a GalNAc residue (A2G1GalNAc1) and a core fucosylated biantennary structure (FA2G2 at 7.5 GU) in a proportion of 60% and 40%, respectively (Fig. 3b).

Core fucosylated PSA *N*-glycans in the UB fraction were evaluated in both total and ABS digested profiles, and slight differences were observed between standard and aggressive PCa PSA. About 40–50% of the *N*-glycan structures were core fucosylated in aggressive PCa blood serum PSA, which represents a lower percentage of core fucosylated structures than in the standard PSA.

On the other hand, the total profile of the B fraction from standard PSA mainly contained disialylated biantennary structures at GUs 9.5 and 10 (Fig. 4a), consistent with the previously reported results^27^. This fraction also showed structures containing GalNAc residues as α2,6/α2,3 disialylated FA2G1GalNAc1S2 glycoforms at 9.1 and 9.5 GUs and FA2G1GalNAc1S1 (α2,6) at 8.3 GU (Fig. 4a), which were digested to a single peak at 7.1 GU (FA2G1GalNAc1) after ABS digestion (Fig. 4b) as previously described in the standard PSA glycan characterisation (Fig. 1).

The most relevant changes in the total profile of the B fractions when comparing blood serum PSA from aggressive PCa patients with standard PSA (Fig. 4a), were a noteworthy decrease of the disialylated core fucosylated glycan structures with α2,6-SA at 10.0 GU, which was the main peak in standard PSA (Fig. 1), and an increase of the structures containing GalNAc: FA2G1GalNAc1S2 (α2,6/α2,3) and FA2G1GalNAc1S1 (α2,6) at 9.1 and 8.3 GUs, respectively. Indeed, most overall PSA glycans in the B fractions of both aggressive PCa and standard PSA were core fucosylated (80–90%) distributed in different glycan structures (Fig. 4).

**Figure 5:**
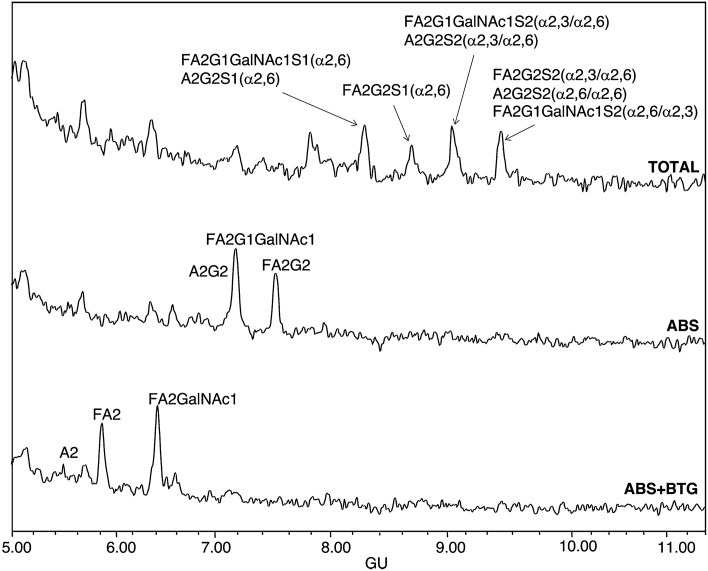
HILIC-UPLC profiles of PSA *N*-glycans from aggressive prostate cancer (PCa6) bound (B) fraction: total profile (top panel), after ABS digestion (middle panel) and after ABS + BTG digestion (bottom panel). Profiles are standardised against a dextran hydrolysate with glucose units (GU).

Furthermore, in order to calculate the proportion of the PSA glycoforms in which GalNAc is present in the B fractions from aggressive PCa blood serum samples, PCa1, PCa5 and PCa6 were further digested with ABS and ABS + BTG (BTG releases β1-3,4 galactose) (Fig. 5). In ABS digestions, FA2G2 (7.5 GU) was separated from FA2G1GalNAc1 and A2G2, but these two last structures co-eluted in a broad peak at 7.2 GU. ABS + BTG digestions allowed to separate FA2G1GalNAc1 and A2G2 structures, which digested to FA2GalNAc1 (7.2 GU) and A2 (5.4 GU) respectively, and therefore the proportions of GalNAc structures and core fucosylated glycans were calculated. In all PSA from aggressive PCa blood samples, approximately 50% of the glycan structures were core fucosylated biantennary structures with LacdiNAc (FA2G1GalNAc1), nearly 40% core fucosylated biantennary with LacNAc (FA2G2) and about 10% non-fucosylated biantennary structures also with LacNAc (A2G2).

The main changes of PSA glycoforms found in aggressive PCa compared with standard PSA are summarised in Table 4.

**Table 4:**
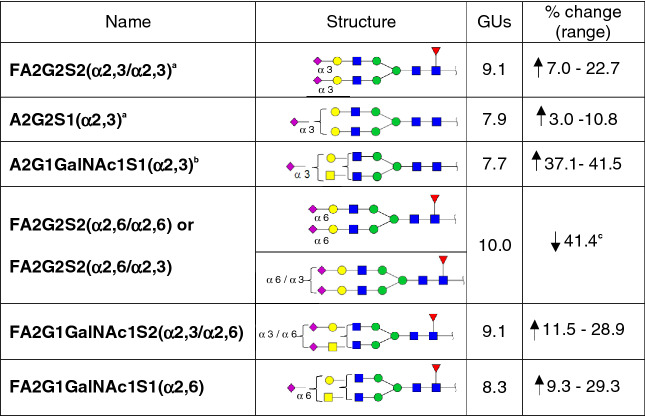
Summary of the differentially expressed PSA glycoforms in aggressive prostate cancer (PCa) blood serum samples.

PSA glycoforms were separated by SNA-chromatography and the percentage of the glycoforms was calculated. The reduction or increase (% change) of each glycoform was calculated by comparing the percentage of the PSA glycoforms from the six PCa blood serum samples with the one of standard PSA.

^a^Changes in this PSA glycoform were only observed in group 1 pattern of the unbound PSA fractions of aggressive PCa samples.

^b^These PSA glycoforms were only present in group 2 pattern of the unbound PSA fractions of aggressive PCa samples.

^c^These PSA glycoforms were not present in any bound PSA fraction of aggressive PCa samples.

## Discussion

Glycosylation is a process altered in PCa^43,44^ and this fact affects the glycosylation of prostate secreted proteins such as PSA^45,46^. In addition, differences in the PSA glycosylation pattern have been found between benign prostate alterations and PCa, and between aggressive PCa and indolent PCa^26–28,30,33,47–49^. In particular, differences in the proportions of PSA fucosylation, sialylation and GalNAc are found in aggressive stages. However, none of these studies have been focused on identifying which specific PSA glycoform or glycoforms are altered in this group of patients. Thus, the aim of this study was to identify the aggressive PCa specific glycoform(s) to further improve PCa risk stratification in the clinics.

The glycosylation analysis of PSA from the blood serum of several aggressive PCa patients performed in this study revealed major differences in their glycan structures when compared to standard PSA. PSA glycans were complex sialylated biantennary structures in both type of samples, but important differences in SA linkage and GalNAc composition were found between standard PSA and aggressive PCa blood serum samples. In particular, the most abundant *N*-glycans were characterised after SNA affinity chromatography. The PSA glycoforms from aggressive PCa patients showed two groups of *N*-glycan patterns in the UB fraction (α2,3-SA glycoforms) different from the ones obtained for the standard PSA; group 1 with FA2G2S2 (α2,3/α2,3) and group 2 with A2G1GalNAc1S1 (α2,3) as the major PSA glycoforms (Table 4). In the SNA affinity chromatography B fractions (α2,6-SA glycoforms), the most prominent difference in PCa samples was the marked reduction of the α2,6-SA disialylated core fucosylated structures (FA2G2S2 (α2,6/α2,6) and FA2G2S2 (α2,6/α2,3)), which corresponds to the main glycoforms in standard PSA. Furthermore, there was an increase of α2,6-sialylated core fucosylated structures containing GalNAc in all the B fractions of PCa samples compared to standard PSA, being the major PSA glycoforms in aggressive PCa (Table 4).

Abnormal sialylation has been associated with poor prognosis and metastasis in cancer^23,24^. An increase of α2,3-SA in PSA was shown in different studies^27–30,32,41^. Several techniques were developed to determine the percentage of α2,3-SA in PCa patients. For instance, Ishikawa et al.^32^ designed an automated microcapillary electrophoresis-based immunoassay system (μTAS system) which was able to detect and quantify the α2,3-SA composition of fPSA by using *Maackia amurensis* agglutinin which is a lectin with high affinity to α2,3-SA. The cut off value to differentiate PCa form BPH patients by α2,3-SA was 43.85% with an area under the curve (AUC) of 0.83, which was higher than the one of tPSA, with an AUC of 0.51. Llop et al.^27^ using SNA affinity chromatography found an increase of the percentage of α2,3-SA in serum PSA in high-risk PCa compared with BPH and indolent PCa. The cut off value of α2,3-SA was set at 30% with a sensitivity and specificity of 85.7% and 95.5%, respectively and with an AUC of 0.97. Afterwards, Ferrer-Batallé et al.^28^ studied the combination of PHI and percentage of α2,3-SA as a new PCa aggressiveness biomarker and the combination of both biomarkers increased the AUC up to 0.99 with a 100% sensitivity and 94.7% specificity. All these studies reported an increase in the percentage of α2,3-SA PSA glycoforms in aggressive PCa, which is consistent with our findings. Thus, we have shown that the percentage of α2,3-SA increases in aggressive stages, mainly due to the increase of FA2G2S2 (α2,3/α2,3) and A2G1GalNAc1S1 (α2,3) glycoforms and the decrease of the FA2G2S2 (α2,6/α2,6) and FA2G2S2 (α2,6/α2,3) glycoforms (see Table 4).

Several studies have shown that the LacdiNAc group is detected in a variety of human cancers and its expression is associated with tumour progression^50^. Specifically in PCa there is an increase of LacdiNAc structures in PSA from PCa blood serum, tissues and also PCa cell lines^29,33,39,49,51,52^. Different quantitative methods with high sensitivity such as surface plasmon-field enhanced fluorescence spectroscopy and mass spectrometry (MS) have been designed to study LacdiNAc structures and to reduce the PCa false-positives of the PSA test. However, the AUC was not significantly higher than that of tPSA as a PCa biomarker^33,51,52^. In agreement with our results, in a recently published study with a cohort of more than 800 patients, the percentage of LacdiNAc residues was found higher in patients with a high Gleason scores. In addition, the AUC of LacdiNAc-PSA (LDN-PSA) (AUC 0.83) was significantly higher than that of tPSA (AUC 0.71). They demonstrated that LDN-PSA in combination with tPSA levels, PSA density and f/t PSA ratio can reduce the number of unnecessary biopsies^34^. In our study, we have validated with a different methodology (HILIC-UPLC) these results and have been able to identify the specific PSA glycoforms containing GalNAc in serum from aggressive PCa. These consisted of different α2,3 and α2,6 glycoforms: FA2G1GalNAc1S2 (α2,6/α2,3) isomers, FA2G1GalNAc1S1 (α2,6) and A2G1GalNAc1S1 (α2,3) (see Table 4).

Another PSA glycan alteration in PCa patients is related to changes in the proportion of core fucosylation. Several authors have described a decrease of core fucose (α1,6-fucose linked to *N*-acetylglucosamine) in serum PSA from aggressive PCa. *N*-glycan sequencing analysis in a few serum samples of aggressive PCa patients with high tPSA showed a significant decrease of core fucose^41,53^. According to these results, Llop et al.^27^ described using PhoSL lectin, a significant decrease of core fucosylation in serum PSA in a cohort of high-risk PCa patients vs. BPH patients with 90% sensitivity and 95% specificity, with an AUC of 0.94. However, other authors have found high levels of PSA glycoforms with core fucosylation in PCa patients with higher Gleason scores using AAL and LCA affinity immunoassay^54^. Recently, Lang et al.^55^ which used MS analyses to quantify core fucosylated glycoforms of PSA, described that the percentage of core fucose glycoforms of PSA shows a decreasing trend in aggressive cancers, but it is not significant enough to improve the distinction between non-aggressive and aggressive PCa or between BPH and PCa. In agreement with this last report, the proportion of core fucosylated structures was very similar in the B fractions of aggressive PCa and standard PSA, but it moderately decreased in the UB fractions of aggressive PCa. The specific glycoforms containing core fucosylation differed between both samples. In particular, there was an increase in the core fucosylated structures with GalNAc, concomitant with a decrease of the core fucosylated FA2G2S2 (α2,6/α2,6) and FA2G2S2 (α2,6/α2,3) in the B fractions of PSA from the aggressive PCa compared with standard PSA. On the other hand, in the PCa UB fractions there were an increase of non-core fucosylated PSA glycoforms, A2G2S1 (α2,3) (in cancer samples with group 1 pattern) and A2G1GalNAc1S1 (α2,3) (in cancer samples with group 2 pattern). However, other UB fractions showed a high percentage of FA2G2S2 (α2,3/α2,3) (in cancer samples with group 1 pattern). As PSA from aggressive PCa samples displays a high percentage of the UB fraction, those cancer samples with group 2 pattern with high proportion of non-core fucosylated structures would show a higher decrease of general core fucose proportions than cancer samples with group 1 pattern. This indicates that PSA from aggressive PCa patients is heterogeneous regarding core fucose proportions. Overall, the moderate reduction of core fucosylation in α2,3-SA glycoforms of PSA in aggressive PCa versus standard PSA would not probably be significant enough to use core fucosylation as a feature to identify PCa aggressiveness.

The molecular mechanisms that lead to aberrant glycosylation of cancer glycoproteins (in this case, PSA glycosylation) are still unclear. The noteworthy changes in sialylation and GalNAc composition that we have described in blood serum PSA from aggressive PCa could be a reflection of changes that occur in the *N*-glycan biosynthesis pathways from prostate cancer cells. Changes in gene expression and enzymatic activity of several glycosyltransferases have been reported in cancer cells and could affect the main sialyltransferases (ST) involved in the addition of α2,6-SA and α2,3-SA: ST6Gal1, ST3Gal3, ST3Gal4 and ST3Gal6, and the *N*-acetylgalactosaminyltransferases B4GALNT involved in the transfer of β1-4 GalNAc^50^.

Concerning the increase of the LacdiNAc group in aggressive PCa, the β4-*N*-acetylgalactosaminyltransferase β4GALNT4, which synthetizes the LacdiNAc group on the outer branches of *N*-glycans has been found upregulated in PCa^34,50,56^. Thus, the β4GALNT4 upregulation in PCa tissues could explain the formation of the LacdiNAc group in FA2G1GalNAc1S2 (α2,6/α2,3) isomers, FA2G1GalNAc1S1 (α2,6) and A2G1GalNAc1S1 (α2,3) PSA glycoforms. In fact, RNA and protein β4GALNT4 levels have been reported to be upregulated in PCa tissues with Gleason scores ≥ 4^34^.

A limitation of this study was the low number of PCa patients’ blood serum samples analysed. In order to structurally characterise the PSA *N*-glycans by *N*-glycan sequencing, at least 1 µg of purified PSA from blood samples was needed. This required the use of blood samples from PCa patients with high levels of PSA in blood serum. Nevertheless, the results obtained have confirmed that the main glycosylation changes found in PSA from the aggressive PCa patients were a rise in glycan structures with α2,3-SA and with GalNAc, and it has revealed the particular PSA glycoforms that are importantly decreased or increased in the aggressive PCa samples (see Table 4). Although the methodology described in this study is not suitable for the routine clinical diagnosis of prostate cancer, the identification of these PSA glycoforms in aggressive PCa patients paves the way to improve the diagnosis of aggressive PCa by designing methodologies addressed to specifically measure those PSA glycoforms, such as imprinted nanoparticles or biosensors^57,58^. These methodologies could be useful to increase sensitivity and specificity in identifying high-risk PCa patients and could be easily translated into clinical diagnosis procedures to facilitate PCa risk stratification.

## Materials and methods

### Blood serum samples

Blood serum samples were from the Hospital Universitari Dr. J. Trueta (Girona, Spain) and were collected following the standard operating procedures of its Ethics Committee, in accordance with the current Declaration of Helsinki, and the European Regulation (EU) 2016/679 and the Spanish Organic Law 3/2018 on data protection. The Ethical approval for this study was obtained from the Comitè d’Ètica d’Investigació Hospital Universitari Dr. J. Trueta (Girona), reference number 388/C/2019. Informed consent was obtained from all patients who provided the human sera. Once collected, sera were stored at − 80 °C. PSA levels (total and free) were quantified using Elecsys PSA electrochemiluminescence immunoassay (ECLIA) in the Modular Analytics E170 (Roche Diagnostics). The Urology and Pathology units from Hospital Universitari Dr. J. Trueta (Girona, Spain) performed the diagnosis using Transrectal Ultrasound-guided biopsy and/or adenomectomy/prostatectomy followed by pathological analysis. The blood serum samples corresponded to six aggressive PCa patients with high levels of total PSA and with a Gleason score ≥ 8. Samples were named from PCa1 to PCa6, and their tPSA levels were: PCa1 (921.7 ng/ml), PCa2 (405.2 ng/ml), PCa3 (311.6 ng/ml), PCa4 (5503.0 ng/ml), PCa5 (1127.0 ng/ml), PCa6 (1481.0 ng/ml).

PSA purified from healthy individuals’ seminal plasma (standard PSA) was from BBI solutions (P117-7) and was used as a control.

### Separation of the α2,3/α2,6-sialic acid PSA glycoforms

Separation of blood serum α2,3 and α2,6-SA PSA glycoforms was performed using SNA chromatography as described previously^27^. Briefly, 0.75 ml of serum samples were treated with 1 M ethanolamine to release the PSA complexed to ACT. The tPSA was immunopurified using paramagnetic particles coated with mouse monoclonal anti-PSA antibody from Access Hybritech PSA assay Kit (Beckman Coulter, Brea, CA, USA), and then desalted and concentrated up to a final volume of 40 µl with Amicon Ultra-0.5–3 K Centrifugal Filter Devices (Millipore, Cork, Ireland). Concentrated samples were processed by lectin affinity chromatography using SNA-agarose lectin (Vector Laboratories, Inc., Burlingame, CA, USA). Eluted unbound and bound fractions were collected by centrifugation and the fPSA of these fractions was quantified by Roche ELECSYS platform to determine the relative percentages of fPSA in the unbound (corresponding to PSA glycoforms containing α2,3-SA), and the bound fraction (containing α2,6-SA PSA glycoforms).

### Immunoprecipitation of fPSA

To purify the PSA glycoforms of the bound and unbound SNA chromatography fractions, fPSA from these collected fractions was immunoprecipitated by using magnetic beads coated with a biotinylated mouse monoclonal antibody anti-fPSA M-30 (Roche Diagnostics) in accordance with a previously published method with some modifications^48^. Briefly, 1 ml of streptavidin coated magnetic beads (0.72 mg/ml) was washed three times with 500 µl of washing buffer (50 mM Tris, 150 mM NaCl, pH 7.4, 1% Triton X-100) and incubated with M-30 antibody for 30 min at room temperature (RT) in incubation buffer (50 mM Tris, 150 mM NaCl, pH 7.4, 0.1% Tween-20, 1% BSA) with shaking. Afterwards, magnetic beads coated with the M-30 antibody were washed three more times and the SNA chromatography fractions were incubated for 1 h at RT with shaking. Beads were washed three times and fPSA was then eluted by adding 45 µl Laemmli buffer 1 × with 5% β-mercaptoethanol and incubated for 30 min at RT under shaking. Finally, the eluted fractions were heated for 5 min at 95 °C.

### Gel electrophoresis analysis of protein fractions

10 µg of standard PSA and the immunoprecipitated PSA fractions from the SNA chromatography were resolved on 12% SDS-PAGE gel under reducing conditions. Each gel was stained for 3 h with 0.25% (w/v) Coomassie Brilliant Blue R-250 in a solution of 50% methanol and 10% acetic acid. Gels were partially destained with solution containing 50% methanol and 7% acetic acid for 5 min at RT and properly unstained overnight using solution containing 5% methanol and 7% acetic acid at RT.

### PSA bands: *N*-glycans release and mass spectrometry (MS) analysis

PSA bands were excised with a scalpel, cut into small pieces (around 1 mm^3^) and frozen at − 20 °C. PSA *N*-glycans were extracted from the gel pieces following the previously reported methodology^42^. Briefly, gel pieces were washed with 20 mM NaHCO_3_ and acetonitrile (MeCN), reduced with 50 mM DTT for 30 min at 60 °C and alkylated with 10 mM iodoacetamide (IAA) for 30 min at RT in the dark. Gel pieces were washed with MeCN and dried in the vacuum centrifuge. PSA *N*-glycans were released by adding 5 µl of N-glycosidase F (PNGase F) (1,000 units/ml, Roche Diagnostics, Mannheim, Germany) diluted with 20 mM NaHCO_3_ or 200 uL PNGase F (500,000 units/ml, New England Biolabs, UK) diluted 1/400 in 20 mM NaHCO_3_ to completely cover the gel pieces and then incubated at 37 °C overnight. Glycans were extracted from gel pieces with cycles of washing using water and MeCN and then, completely dried.

### In-gel trypsin digestion and MS analyses of the PSA bands

After PNGase F digestion and glycans extraction, gel pieces were washed with 100 µl H_2_O for 15 min and dehydrated with 100 µl of MeCN for 15 min, solvent was removed and then gels were totally dried in a SpeedVac vacuum concentrator. Gel pieces were digested with 100 µl of trypsin solution (0.20 ng/l in 50 mM NH_4_HCO_3_ pH:7.8) initially for 30 min at RT, followed by the addition of 50 mM NH_4_HCO_3_ to top up the gels, and incubated overnight at 37 °C. Samples were spinned and supernatants were collected. Then peptides were extracted again by one wash with 100 µl H_2_O and three washes of 100 µl 0.1% trifluoroacetic acid (TFA), 50% MeCN for 20 min each in the ultrasonic bath. The supernatants of each sample washes were combined with the initial supernatant and were evaporated in a SpeedVac vacuum concentrator to 10 µl. Before the analyses, 1 µl of 1% TFA was added to the samples and then 1 µl of each sample were mixed with 1 µl of α-cyano-4-hydroxycinnamic acid matrix 10 g/l in MeCN/H_2_O/TFA (80:20:0.1 v/v/v), and the mixture was spotted on the MALDI plate (AnchorChip target plate 400/384 TF, Bruker Daltonics, Bremen, Germany) and allowed to dry at RT. Experiments were carried out on an Autoflex MALDI-TOF (Bruker Daltonics). Samples were measured in reflectron mode with positive polarity. Internal manual calibration was performed using Peptide Calibration Standard II from Bruker Daltonics. Several spectra were collected from random points for sample and each spectrum was obtained with 500 laser shots per point. Recorded data were processed with flexanalysis software (version 3.4) from Bruker Daltonics. Peptides from mass spectra were matched against Swiss-Prot database using the Mascot search engine (Matrix Science).

### *N*-Glycan sequencing of PSA glycans

Subsequently, the obtained *N*-glycans were fluorescently labelled with 10 µl of 2-aminobenzamide (2AB) for 2 h at 65 °C. For each sample, 1 cm^2^ of a chromatography paper (3MM Whatman) was used to eliminate the excess of 2AB reagent through MeCN washing in Fisherbrand^™^Polypropylene 96 well microplates, and *N*-glycans were finally eluted with water and dried in the vacuum centrifuge as described^42^.

The 2AB-labelled *N*-glycans from PSA were analysed by UPLC with fluorescence detection on a Waters Acquity UPLC H-Class system consisting a quaternary solvent manager, sample manager and fluorescence detector under the control of Empower3 software (Waters, Mildford, MA, USA). The *N*-glycans were separated by HILIC using Acquity UPLC-BEH Glycan column 2.1 × 150 mm, 1.7 µm BEH particles. As previously described^27,59^, solvent A was 50 mM formic acid adjusted to pH 4.4 with ammonia solution and solvent B was MeCN. The column temperature was set to 40 °C. A 30 min method was used with a liner gradient 70–53% MeCN at 0.56 ml/min. The injection volume was 20 µl of sample prepared in 70% (v/v) MeCN. The fluorescence detection excitation/emission wavelengths were λex = 330 nm and λem = 420 nm, respectively. The retention time from the resulting profiles were standardised with a dextran ladder to glucose units (GUs).

### Exoglycosidase digestions of PSA *N*-glycans

The 2AB-labelled glycans were digested at 37 °C for 16 h using several exoglycosidases from Prozyme, either alone or in an array: 0.5 U/ml ABS (α2-3,6,8,9 Neuraminidase A, digests all sialic acids), 5 U/ml NAN1 (α2-3 Neuraminidase S, digests α2-3 linked *N*-acetylneuraminic acid residues), 1 U/ml BTG (β1-3,4 Galactosidase, digests β1-3,4 galactose), 1 U/ml BKF (α1-2,3,4,6 Fucosidase, digests α1-2,3,4,6 fucose), 8 U/ml GUH (β-*N*-Acetylglucosaminidase S, digests β-*N*-acetylglucosamine) and 10 U/ml JBH (Jack bean-*N*-acetylhexosaminidase, digests β *N*-acetylglucosamine and β1,2,3,4,6 *N*-acetylgalactosamine). After digestion, enzymes were inactivated at 65 °C and removed by filtration in 10 K microcentrifuge filtration devices (Pall 516-8491). The chromatography profiles were obtained as described in the *N*-glycan sequencing part.

## Supplementary information

Supplementary Legends.

Supplementary Figure 1.

Supplementary Figure 2.
