# Supplementary material for: Characterisation of the main PSA glycoforms in aggressive prostate cancer
Source: Sci Rep. 2020 Nov 4;10:18974. doi: 10.1038/s41598-020-75526-3 (PMC7643140; doi:10.1038/s41598-020-75526-3)
Supplement: Supplementary file 1 — Supplementary Legends. [file 41598_2020_75526_MOESM1_ESM.docx]

**Characterisation of the main PSA glycoforms in aggressive prostate cancer**

Anna Gratacós-Mulleras^1,2^, Adrià Duran^1,2^, Akram Asadi Shehni^3^, Montserrat Ferrer-Batallé^1,2^, Manel Ramírez^2,4^, Josep Comet^2,5^, Rafael de Llorens^1,2^, Radka Saldova^3,6^, Esther Llop^1,2^, Rosa Peracaula^1,2^.

^1^ Biochemistry & Molecular Biology Unit. Department of Biology, University of Girona, Girona, Spain.

^2^ Girona Biomedical Research Institute (IDIBGI), Girona, Spain.

^3^ NIBRT GlycoScience Group, National Institute for Bioprocessing Research and Training, Dublin, Ireland.

^4^ Clinic Laboratory, Dr. J. Trueta University Hospital, Girona, Spain.

^5^ Urology Unit, Dr. J. Trueta University Hospital, Girona, Spain.

^6^ UCD School of Medicine, College of Health and Agricultural Science (CHAS), University College Dublin (UCD), Dublin, Ireland.

**Supplementary figure legends:**

**Supplementary figure 1.** Gel electrophoresis of standard PSA immunoprecipitated from unbound (UB) and bound (B) fractions of the SNA chromatography. A molecular weight marker (MW marker) was loaded in the first lane.

**Supplementary figure 2.** Gel electrophoresis of PSA from prostate cancer blood serum sample 4 (PCa4) immunoprecipitated from unbound (UB) and bound (B) fractions of the SNA chromatography. A molecular weight marker (MW marker) was loaded in the first lane.
