# Supplementary figures and images for: Characterisation of the main PSA glycoforms in aggressive prostate cancer
Source: Sci Rep. 2020 Nov 4;10:18974. doi: 10.1038/s41598-020-75526-3 (PMC7643140; doi:10.1038/s41598-020-75526-3)

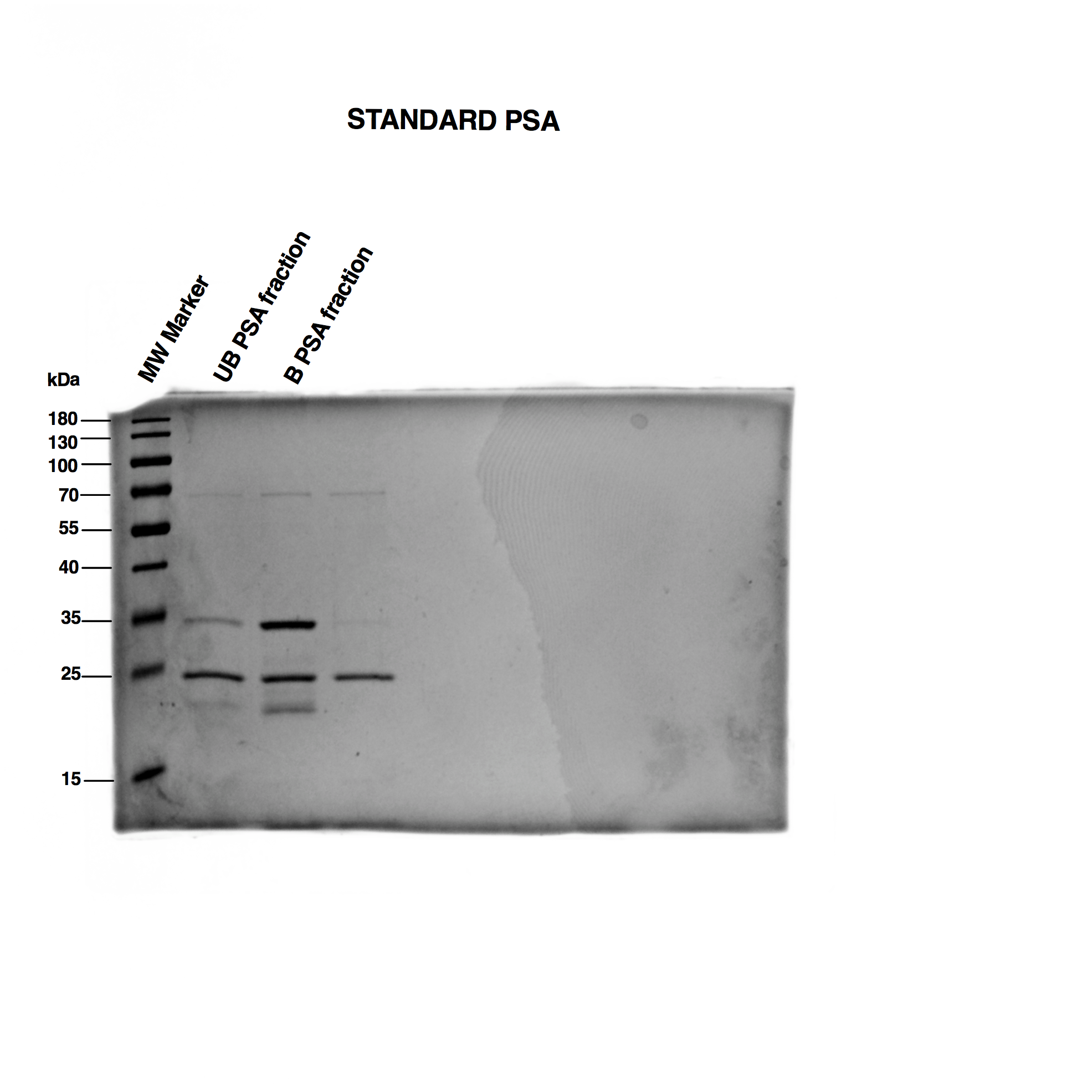

Supplement: Supplementary file 2 — Supplementary Figure 1. [file 41598_2020_75526_MOESM2_ESM.tiff]

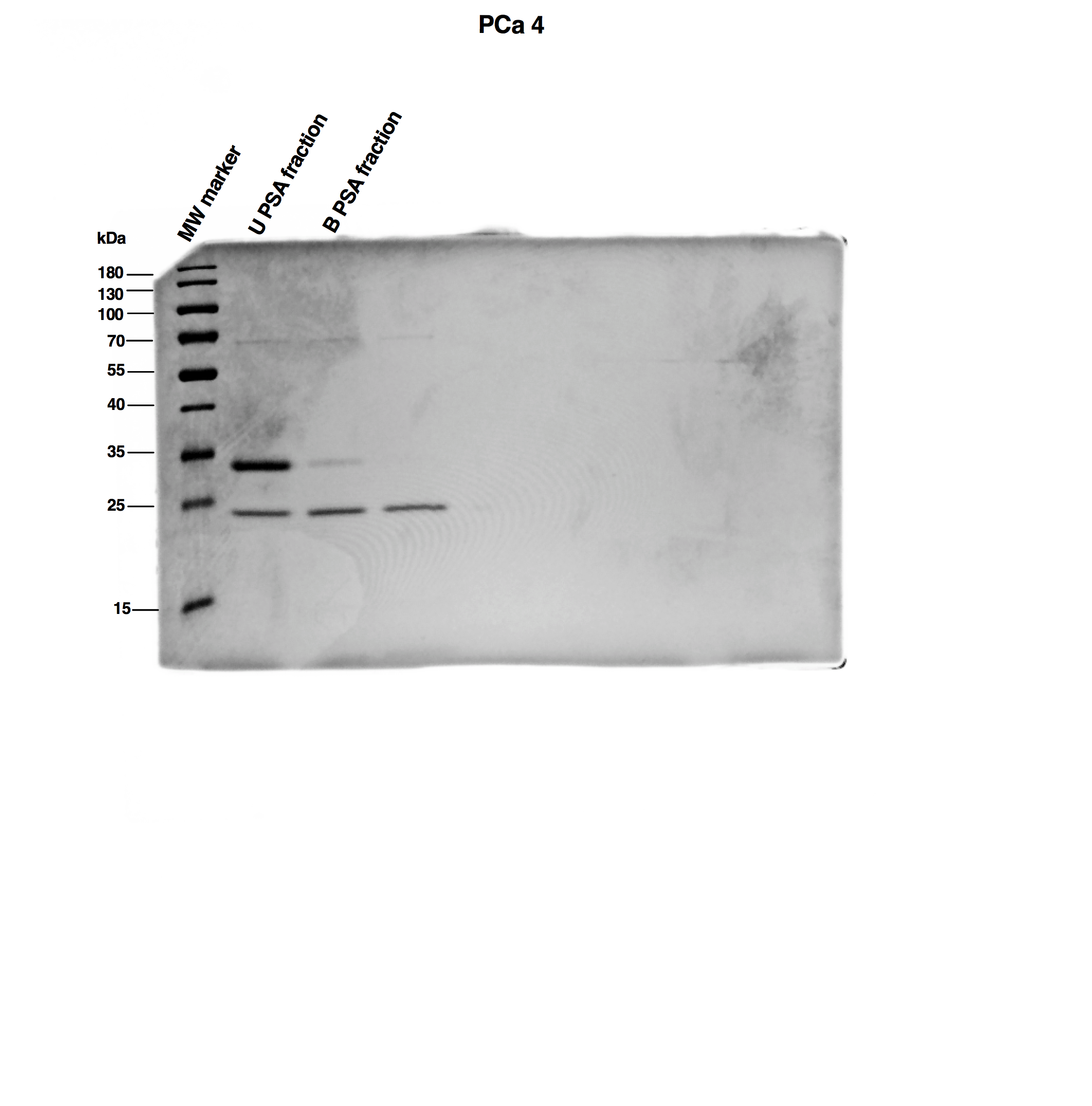

Supplement: Supplementary file 3 — Supplementary Figure 2. [file 41598_2020_75526_MOESM3_ESM.tiff]
